# Safety and efficacy of 650 nm invasive laser acupuncture on non-specific chronic low back pain: A protocol for a multicenter randomized placebo-controlled trial

**DOI:** 10.3389/fmed.2023.1021255

**Published:** 2023-02-09

**Authors:** Jae-Hong Kim, Changsop Yang, Jaehee Yoo, Gwang-Cheon Park, Byoung-Kab Kang, Ae-Ran Kim, Jihye Kim, Dongwoo Nam, Yejin Hong

**Affiliations:** ^1^Department of Acupuncture and Moxibustion Medicine, College of Korean Medicine, Dongshin University, Naju, Republic of Korea; ^2^Clinical Research Center, Dongshin University Gwangju Korean Medicine Hospital, Gwangju, Republic of Korea; ^3^KM Science Research Division, Korea Institute of Oriental Medicine, Daejeon, Republic of Korea; ^4^Clinical Research Coordinating Team, Korea Institute of Oriental Medicine, Daejeon, Republic of Korea; ^5^Digital Health Research Division, Korea Institute of Oriental Medicine, Daejeon, Republic of Korea; ^6^Department of Acupuncture and Moxibustion, College of Korean Medicine, Kyung Hee University, Seoul, Republic of Korea; ^7^Department of Acupuncture and Moxibustion, Kyung Hee University Korean Medicine Hospital, Seoul, Republic of Korea

**Keywords:** safety, efficacy, non-specific chronic low back pain, study protocol, randomized controlled trial, laser acupuncture

## Abstract

**Background:**

We aim to obtain clinical trial data regarding the safety, efficacy, and usefulness of invasive laser acupuncture (ILA) for non-specific chronic low back pain (NSCLBP) through a randomized placebo-controlled trial.

**Methods:**

Our clinical trial will be an assessor- and patient-blinded, prospective, parallel-arm, multi-center, randomized placebo-controlled clinical trial. One hundred and six participants with NSCLBP will be allocated evenly to the 650 ILA or control group. All participants will receive education on exercise and self-management. The 650 ILA group will undergo 650 nm ILA for 10 min, and the control group will undergo sham ILA for 10 min per visit, twice a week for 4 weeks, at bilateral GB30, BL23, BL24, and BL25. The primary outcome will be the proportion of responders (≥30% reduction in pain visual analogue scale [VAS] without increased use of painkillers) at 3 days after the intervention ends. The secondary outcomes will include changes in the scores of the VAS, European Quality of Life Five Dimension Five Level scale, and Korean version of the Oswestry Disability Index at 3 days after the intervention ends and 8 weeks after the intervention ends.

**Discussions:**

The results of our study will provide clinical evidence concerning the safety and efficacy of 650 nm ILA for the management of NSCLBP.

**Clinical trial registration:**

https://cris.nih.go.kr/cris/search/detailSearch.do?search_lang=E&focus=reset_12&search_page=M&pageSize=10&page=undefined&seq=21591&status=5&seq_group=21591, identifier KCT0007167.

## Introduction

Low back pain (LBP), defined as discomfort and pain, localized below the costal margin and above the inferior gluteal folds with or without referred lower extremity pain, is a symptom rather than a specific disease (1, 2). It is a major cause of disability in daily living. Functional restrictions and consequent disabilities pose a huge economic burden for individuals and the society (3). Most LBPs are non-specific (commonly cited as 90–95%) (4), and non-specific chronic LBP (NSCLBP) is defined as that which cannot be attributed to a known specific pathology (e.g., structural deformity, tumor, osteoporosis, infection, radicular syndrome, fracture, cauda equina syndrome, or ankylosing spondylitis) and persists for >3 months (1, 2, 5–7).

Treatment guidelines for patients with NSCLBP recommend the use of exercise therapy, antidepressants, psychosocial interventions, and non-steroidal anti-inflammatory drugs (2, 5, 6). Non-pharmacological treatments, such as exercise and self-management, are preferred to pharmacological interventions in the management of NSCLBP (2, 7–9). Physical modalities such as transcutaneous electrical nerve stimulation and low level laser therapy (LLLT) have a low quality evidence (6, 8) and acupuncture has mixed support and received conflicting recommendations (2, 5, 8).

Low level laser therapy is a light source intervention that creates no vibration, heat, or sound and stimulates photochemical or non-thermal cellular processes (10). LLLT is currently used to treat musculoskeletal disorders, such as back pain (11). The possible underlying mechanisms for the pain reduction effects of LLLT include its inhibition of neural function, anti-inflammatory effect, and connective tissue repair ability which have been demonstrated by a number of experiments (12–14). Laser acupuncture (LA) is the irradiation of a low-intensity laser at the acupoints using laser pointer devices (15). Whether LLLT, including LA, is effective for LBP when compared with sham laser remains controversial. A Cochrane systematic review of LLLT on non-specific LBP in 2008 decried the lack of sufficient data to either refute or support the efficacy of LLLT for the management of LBP (16). In contrast, a meta-analysis suggested that LLLT was an effective treatment for reducing pain in patients with NSCLBP (17, 18). Glazov et al. reported that LLLT alone or in combination with other therapies might effectively reduce pain for up to 3 months in NSCLBP without adverse effects (19).

While LA is a non-invasive therapy that uses laser-emitting devices, invasive LA (ILA) is conducted concurrently with invasive acupuncture and focused laser irradiation utilizing an acupuncture needle attached to a laser instrument (20). Our previous pilot randomized controlled trial (RCT) revealed that 650 nm ILA with the same ILA parameters (650 nm wavelength, 20 mW power, and 50 Hz frequency), treatment acupoint, and treatment schedule as in this study significantly improved pain and pain-related functional limitations in patients with NSCLBP at the end of the intervention (21). These potential effects of 650 nm ILA on NSCLBP requires validation using a rigorous RCT with a large sample size. Therefore, we aim to obtain clinical evidence on the safety and efficacy of 650 nm ILA for the management of NSCLBP. In a situation where is no high-quality evidence supporting the efficacy of LLLT and acupuncture, the results of this study will provide clinical evidence of the use of ILA for NSCLBP and thus promote the use of ILA in the treatment of NSCLBP.

## Materials and analysis

### Aims

1) We will investigate the clinically persistent pain-reduction effect of 650 nm ILA for NSCLBP at 3 days after the end of intervention.
2) We will investigate the pain reduction, functional limitation improvement, and quality of life improvement effects of 650 nm ILA for NSCLBP.
3) We will investigate the safety of 650 nm ILA in patients with NSCLBP.

### Hypothesis

1) The 650 ILA group will have a significantly higher proportion of responders than the control group at 3 days after the end of intervention.
2) The 650 nm ILA will show improvements in pain intensity, quality of life, and functional limitation in patients with NSCLBP.
3) The 650 nm ILA would be a safe treatment for patients with NSCLBP.

### Study design and setting

Our study was approved by the Ministry of Food and Drug Safety (Medical Device Approval No. 1322) and was registered with the Clinical Research Information Service (registration No. KCT0007167). Our study complies with the Korean Good Clinical Practice guidelines and the principles of the Declaration of Helsinki. We have written the manuscript in accordance with the Standard Protocol Items: Recommendations for Interventional Trials (SPIRIT) reporting checklist (22).

This clinical trial will be an assessor and patient–blinded, prospective, parallel-arm, multi-center, randomized placebo-controlled clinical trial. In total, 106 eligible participants will be randomized evenly into the 650 ILA or control group (*n* = 53 in each group). All participants will receive education on exercise and self-management. Participants in the 650 ILA group will undergo real 650 nm ILA for 10 min, while those in the control group will undergo sham ILA for 10 min. The treatment will be administered once per visit, twice a week for 4 weeks, at bilateral Gallbladder 30 (GB30; Huantiao), Bladder 23 (BL23; Shenshu), Bladder 24 (BL24; Qihaishu), and Bladder 25 (BL25; Dachangshu).

The primary outcome will be the proportion of responders (≥30% reduction in pain visual analogue scale [VAS] without increased use of painkillers) at 3 days after the intervention ends. The secondary outcome will be changes in the scores of the VAS, European Quality of Life Five Dimension Five Level scale (EQ-5D-5L), and Korean version of the Oswestry Disability Index (ODI) at 3 days after the intervention ends and 8 weeks after the intervention ends.

The clinical trial design is shown in Table 1.

**TABLE 1:** SPIRIT statement showing the enrollment, interventions, and data collection.

	Study period							
	Enrollment	Allocation	Post-allocation					Close-out
Timepoint	Screening		Visit 1–2	Visit 3–4	Visit 5–6	Visit 7–8	Visit 9	Visit 10
	Week		1	2	3	4	4 + 3 days	12
**Enrollment**								
Informed consent	X							
Sociodemographic profile	X							
Medical history	X							
Vital signs	X	X	X	X	X	X	X	X
Inclusion/Exclusion criteria	X							
Allocation		X						
Visual analogue scale	X							
**Interventions**								
Invasive laser acupuncture (sham or 650 nm)			X	X	X	X		
Education on self management and exercise			X	X	X	X		
**Assessments**								
Change of medical history			X	X	X	X	X	X
Safety assessment (Incidence of AEs)			X	X	X	X	X	X
Clinical laboratory test	X						X	
Visual analogue scale			X				X	X
European quality of life five dimension five level scale			X				X	X
Scores for the Korean version of the Oswestry disability index level scale			X				X	X

### Recruitment

We will recruit participants at the Kyung Hee University Korean Medicine Hospital and Dongshin University Gwangju Korean Medicine Hospital in the Republic of Korea *via* the use of posters, local newspapers, and the internet in hospitals and communities. Interested people will receive an explanation of this study from the clinical research coordinator (CRC) upon visiting the hospital and will provide written informed consent prior to participation.

At each visit, the CRC will explain the following visit schedule and will adjust the visit schedules for each participant to facilitate participation.

### Inclusion criteria

(1) Adults aged between 19 and 70 years; (2) has NSCLBP lasting for at least the preceding 3 months, and LBP that occurred more than 14 days a month; (3) has no change in history of medications for 4 weeks before screening. For participants with medications prescribed and used for NSCLBP, usage shall be at a stable dose in the 4 weeks prior to the baseline visit; (4) moderate pain (100 mm VAS scores for pain had a range of 35–74) (23) at screening; and (5) has sufficient fluency in Korean to perform valid assessments.

### Exclusion criteria

(1) Participants has progressive neurological deficits or radicular pain; (2) has a serious disease (diabetic neuropathy or cancer, severe kidney, liver, cerebrovascular, or cardiovascular disease); (3) has a serious spinal pathology (cauda equina syndrome, cancer, inflammatory spondylitis, recent vertebral fracture, or spinal infection); (4) has a LBP caused by rheumatoid arthritis, ankylosing spondylitis, gout, trauma, or fibromyalgia; (5) has a history of treatment for mental illness (depression, dementia, schizophrenia, or epilepsy) or drug/alcohol dependency in the 6 months preceding screening; (6) has moderate or severe depression (scored ≥ 23 points on a Korean version of Beck depression inventory-II) (24) at screening; (7) has contraindications for ILA, such as presence of electronic medical devices, severe skin disease in the lumbar region, blood clotting abnormalities, or presence of metallic devices in the lumbar vertebrae; (8) has a history of lumbar spinal surgery within 1 year or scheduled procedures during the trial; (9) participation for the purpose of social insurance or compensation; (10) concurrent participation in another trial; (11) Pregnant or having a plan for pregnancy; and (12) not suitable for ILA and our rescue regimen.

### Dropout and violation criteria

The dropout criteria are as follows: (1) incomplete data that can affect the results of the trial; (2) decision to discontinue participation in this trial by the institutional review board (IRB) or principal investigator (PI) owing to the inability of the participant to participate in this study or the occurrence of a side effects requiring long-term treatment; (3) withdrawal of consent; or (4) occurrence of a serious adverse event (SAE) causing requirement of hospitalization or surgery, serious disability, or death. Participants who meet the dropout criteria will be discontinued from participating in our clinical trial.

The violation criteria are as follows: (1) participating in less than six of the eight treatment sessions (<75% compliance with the intervention protocol); and (2) serious deviation in implementation or critical errors in the protocol.

The participants who meet the violation and dropout criteria will be excluded from the per protocol set (PPS) analysis.

### Ethics

This protocol (version. 1.0) was approved by the Ministry of Food and Drug Safety (date: March 16, 2022; Medical Device Approval # 1322). It was approved by the IRB of Kyung Hee University Korean Medicine Hospital (date: April 22, 2022; approval No.: KOMCIRB 2022-03-004-001) and Dongshin University Gwangju Korean Medicine Hospital (date: March 22, 2022; approval No.: DSGOH-2022-002). Participants and their companions will be informed of the study purpose and risks. All participants will provide written informed consent prior to participation.

### Randomization and allocation

The investigator will carry out a screening interview, and then the assessor will conduct baseline assessment. The 106 enrolled participants will be randomly allocated evenly to the 650 ILA or control group (*n* = 53 per group). The serial numbers will be generated by stratified block randomization using SAS^®^ version 9.4 (SAS Institute Inc., Cary, NC, USA). The serial numbers will be packed in opaque envelopes and stored in a cabinet with two locks.

The investigator managing the serial numbers will open the envelopes and assign participants to the investigator who will conduct the intervention.

### Implementation

The investigator managing the serial numbers will generate the randomization sequence and distribute participants to the groups. The CRC will enroll the participants.

### Blinding

The practitioner will insert the acupuncture needles into GB30, BL23, BL24, and BL25 and then operate the laser emitting device according to the treatment method of each group (650 ILA group, 20 mW power for 10 min; control group, 0 mW power for 10 min). Therefore, the practitioner who will conduct the intervention would know the group assignment of participant. Owing to the practitioner unblinding, we will adopt an assessor and patient-blinded design using sham ILA. During the course of the study, all investigators except those who will perform the intervention and manage the serial number codes will be blinded. However, if necessary, such as in the event of SAE, unblinding will be permitted under the IRB approval. This study will only include individuals without predetermined positions or competing interests.

### Interventions

**FIGURE 1:**
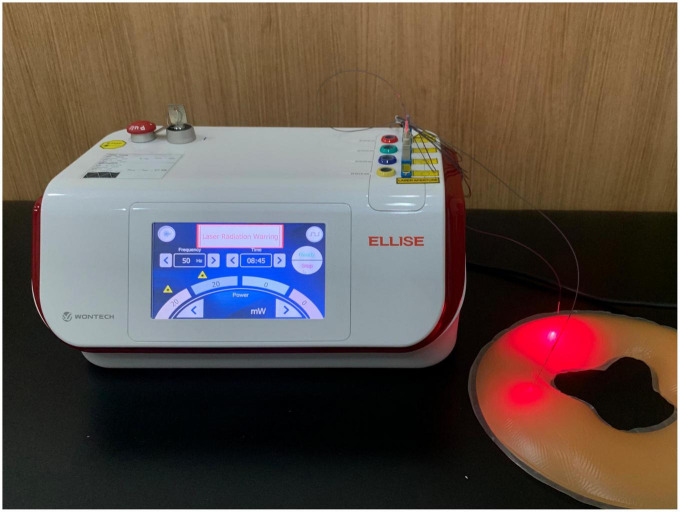
Invasive laser acupuncture (ILA) therapy.

Trained Korean medical doctors will administer the treatment. The investigators who will carry out the interventions will undergo joint training to ensure adherence to our protocol. ILA treatment will be conducted using a laser emitting device (Ellise; Wontech Co. Ltd., Daejeon, Republic of Korea) comprising an optical fiber-coupled laser diode (InGaAIP), a sterile acupuncture needle with optical fibers inserted therein, and a laser output device (Figure 1).

The acupuncture needles will be vertically inserted into GB30, BL23, BL24, and BL25 and then the laser (650 ILA group, 20 mW power for 10 min; control group, 0 mW power for 10 min) will be turned on. The ILA parameters will be 50 Hz frequency, 20 mW power, 63.69 W/cm^2^ power density, 12 J/point energy dose, 38216.56 J/cm^2^ energy density, and pulse type wave. Participants will receive treatment for 10 min per visit, twice a week for 4 weeks. Based on previous RCTs investigating the efficacy of LLLT for NSCLBP (25, 26), the control group will receive the same procedure as the 650 ILA group. No significant differences in sound, feeling, or observation will be expected between the two groups. Hence, all participants will be blinded. The ILA treatment methods have been described in more details in our previous pilot study (20).

All participants will receive education on exercise and self-management during the treatment period (Visit 1–Visit 8). We will provide all participants with acetaminophen (500 mg), which can be taken when the pain is severe as a rescue regimen.

At each visit, the medical condition of the participants will be monitored to ensure that they adhere to the trial procedure. All participants will be expected to comply with the intervention protocol. However, the assessment and treatment schedule may be changed upon the request of the participant or in accordance with the judgment of the PI.

During the study period, all participants will not be permitted to receive other treatments (complementary and alternative therapies, physical therapy, or pharmacological treatments not allowed in our trial) for improving NSCLBP symptoms. However, they will be allowed to use pharmacological and non-pharmacological treatments for improving other symptoms.

### Outcome measurements

The primary outcome will be the between-group differences of the proportions of responders at 3 days after the intervention ends. A responder is defined as a participant who responds with more than 30% decrease in baseline VAS score without an increase in baseline use of painkillers (27, 28).

The secondary outcomes will include the between-group differences of changes in VAS, EQ-5D-5L, and ODI at 3 days after the treatment ends and 8 weeks after the treatment ends.

The VAS is a self-reported scale, usually a 100-mm-long straight line marked 0 for no pain and 100 for pain as intense as it could be (29). It is widely used to assess pain severity in LBP trials (30).

The EQ-5D is a generic assessment tool for measuring health-related quality of life (31). The EQ-5D-5L is a new version of the EQ-5D that extends the level of each dimension from three to five (32).

The ODI includes nine questions about interference with several physical activities and one question about pain intensity (33). We used the Korean version of the ODI, which excluded sexual life from the original ODI and has been validated (34).

### Adverse events

Adverse events (AEs) are unintentional and undesirable symptoms, signs, or diseases that appear during or after treatment in a clinical trial. In our pilot study, no AEs and SAEs were associated with laser irradiation (21). AEs that could occur in our trial include bleeding, pallor, local hematoma, skin irritation, objective worsening of pain, and dizziness or fainting. All the AEs and SAEs would be recorded in detail, including the potential causalities between the intervention and AE, degree of severity, time of occurrence, and any measures taken to improve AE by the CRC. They will be reported to the IRB. The occurrence rate of SAEs and AEs will be compared between the two groups in the safety assessment. Participants who will experience AEs and SAEs related to our intervention will be compensated according to the relevant regulations.

### Quality control

Our protocol has been developed and revised severally by experts in LA, NSCLBP, and statistics. Before the study, all investigators will be trained severally to fully understand the standard operating procedures (SOPs) of this trial and the protocol. An independent clinical research associate will monitor our study and check all the trial documents. Any revision of this protocol will be reviewed and approved by the Ministry of Food and Drug Safety and the IRB of Kyung Hee University Korean Medicine Hospital and Dongshin University Gwangju Korean Medicine Hospital.

### Sample size estimation

In our previous pilot study (21), the difference in proportion of responders between 650 ILA (93% [14/15]) and control groups (40% [6/15]) was 53%. To obtain sufficient clinical data, we determined the sample size assuming an expected responder proportion of 30% in the control group and 60% in the 650 ILA group, a two-sided alpha level of 0.05, and a statistical power of 0.8. Under these assumptions, a total of 84 (42 per group) participants will be required. Estimating a maximum dropout rate of 20%, 106 participants (53 in each group) will be required in our trial.


$$\frac{n={\left(z_{\alpha /2}\,\sqrt{2\,\bar{p}\,\bar{q}}+z_{\beta }\,\sqrt{p_{1}\,q_{1}+p_{2}\,q_{2}}\right)}^{2}}{{\left(p_{t}-p_{c}\right)}^{2}}=\frac{{\left(1.96\,\sqrt{2\times 0.45\times 0.55}+0.842\,\sqrt{0.30\times 0.70+0.60\times 0.40}\right)}^{2}}{{\left(0.60-0.30\right)}^{2}}\approx 42$$

### Statistical analysis

The final data will be analyzed by a biostatistician not involved in the execution of our trial. The primary analysis population for assessing the efficacy of the intervention will be a full analysis set (FAS), while the supplementary analysis population will be a PPS. The results of the FAS and PPS analyses will be compared and reflected in the efficacy evaluation. “Multiple Imputation” method will be used to obtain the missing value. All analyses will be performed at the 5% (two-sided) significance level using SAS^®^ version 9.4 (SAS institute. Inc., Cary, NC, USA) software. We will not perform interim analyses.

Baseline characteristics and variables will be compared between the two groups. Categorical data will be compared using the Fisher’s exact test or chi-square test, while continuous data will be compared using the Wilcoxon’s rank sum test or independent *t*-test.

The difference in responder proportions will be tested using the chi-square test or Fisher’s exact test. The degrees of changes in the VAS, EQ-5D-5L, and ODI scores at 3 days after the treatment ends and 8 weeks after the intervention ends, relative to the baseline score, between the groups will be evaluated using analysis of covariance with baseline scores as covariates. Within each group, changes in VAS, EQ-5D-5L, and ODI scores at each time will be analyzed using one-way analysis of variance and a paired *t*-test or Wilcoxon signed rank test. Sub-analyses will be performed according to each institution.

A safety assessment will be conducted for all SAEs and AEs that will occur during the trial period. The incidences of SAEs and AEs will be compared between the two groups using chi-square test or Fisher’s exact test. A comparative analysis will be carried out between the groups for participants who will be out of normal range for a clinical laboratory test.

### Confidentiality and data management

All documents will be classified and logged with identification codes, but the names will remain concealed.

All identification records will be kept confidential and will not be accessible without IRB approval. All data will be recorded in the electronic case report forms (eCRFs) by the CRC and checked by a investigator, who will not be involved in the execution of this trial. Electronic data will be securely stored in the eCRFs using the myTrial data management system (NIKOM, Republic of Korea). Data access will be protected by user name and password. The data manager will have online access to the all data, whereas institutions will have access only to data related to their own institution. The data manager and the statistician will have online access to the entire database. The data coordinating center in the Korea Institute of Oriental Medicine will be unaffiliated with the sponsor and devoid of competing interests. No access to the data will be granted to anyone not approved by the IRB. In addition, raw data will be stored for 3 years after the completion of the trial. Participants will voluntarily offer informed written consent for the dissemination of their personal information.

## Discussion

The efficacy of LA for musculoskeletal pain is majorly determined by the energy dosage applied (35). Energy penetration through the skin is affected by the scatter, reflection, and absorption of energy by skin structures. As the acupoints are thought to be located in the myofascial layer, the low energy transmission of non-invasive LA may not be fully effective in stimulating acupoints (15). However, the 650 nm wavelength ILA used in this study is safe and can compensate for the scatter, reflection, and absorption of light by the skin and enhance energy transmission, because the laser is emitted at the acupuncture needle’s tip after being placed beneath the skin. The design of this study, including the ILA intervention (i.e., treatment acupoint and laser wavelength), treatment schedules, outcome measurements, and sample size, is based on that of our previous pilot study (21). In our previous pilot clinical trial (21), 650 nm ILA at bilateral GB30, BL23, BL24, and BL25 showed significant improvement in the VAS and ODI scores at the intervention endpoint and ODI score at 4 weeks after the intervention ends, compared with sham laser in patients with NSCLBP. Therefore, BL23, BL24, BL25, and GB30 were selected for treatment acupoints and 650 nm for laser wavelength in this study.

The clinical assessments used in LBP trials are often highly subjective and correlate poorly with symptoms. The responder index could be sensitive to clinically meaningful treatment effects and could mitigate the placebo effect (27). The preliminary developed responder index for chronic low back pain was at least 30% improvement in pain, with an improvement of at least 30% in patient global assessment and no worsening in function (27). We adopted responder proportions (≥30% relief on the VAS without analgesics increase) used in a recent chronic LBP clinical trial as a primary outcome (28). Four core outcome domains for LBP trial are pain intensity, number of deaths, health-related quality of life, and physical functioning (36, 37). We used change in VAS scores to measure the pain intensity, ODI scores to measure the physical functioning, and EQ-5D-5L scores to evaluate the health-related quality of life.

Considering the results of this study, 650 nm ILA is expected to show safety, clinically significant improvement, pain reduction, and improvement of functional limitation and quality of life in patients with NSCLBP. This is important for the clinical use of 650 nm ILA and development of optimal laser parameters for the treatment of NSCLBP.

This protocol has certain limitations. First, we will not use different treatment methods of LA for NSCLBP treatment. The factors that influence the efficacy of LA are selected acupoints, wavelength, and energy dose. Various treatment methods of LA differ according to wavelength, energy dose, and acupoints for treating NSCLBP (16–19, 38). Since 650 nm ILA parameters, including the wavelength, power, energy dose, and acupoints, used in our pilot study show significant pain reduction at the intervention endpoint, we adopted only the 650 nm ILA parameters used in our pilot study in this study. Thus, further studies should be conducted to investigate the optimal parameters. Second, since most participants in our pilot study had moderate pain at the baseline and the high dropout rate owing to pain increase during the study period in the case of patients with severe pain was concerning, our study will include only patients with moderate NSCLBP, not those with severe NSCLBP. Third, since the practitioner will operate the laser emitting device according to the treatment method of each group (650 ILA group, 20 mW power for 10 min; control group, 0 mW power for 10 min), we cannot adopt practitioner blinding. Therefore, we will adopt an assessor- and patient-blinded study design.

Nevertheless, the findings of this study would suggest clinical evidence concerning the safety and efficacy of 650 nm ILA for the management of NSCLBP, thereby establishing the basis for further investigation. It would contribute to increasing the availability of laser and promoting the development of optimal laser treatment method in the management of LBP.

## Ethics statement

This protocol (version. 1.0) was approved by the Ministry of Food and Drug Safety (date: March 16, 2022; Medical Device Approval # 1322). It was approved by the IRB of Kyung Hee University Korean Medicine Hospital (date: April 22, 2022; approval No.: KOMCIRB 2022-03-004-001) and Dongshin University Gwangju Korean Medicine Hospital (date: March 22, 2022; approval No.: DSGOH-2022-002). Participants and their companions will be informed of the study purpose and risks. All participants will provide written informed consent prior to participation.

## Author contributions

J-HK and CY were responsible for designing and conceiving the trial, preparing the manuscript, supervising the entire clinical trial process, and writing and revising the final manuscript. J-HK, JY, G-CP, A-RK, JK, DN, and YH participated in the data collection and were in charge of the recruitment, treatment, and evaluation of the patients. B-KK was responsible for planning the data analysis and analyzing final data from the trial. All authors reviewed and approved the final manuscript.
